# Exercise Interventions and Attentional Performance in Children and Adolescents: Evidence from Randomized Controlled Trials

**DOI:** 10.3390/sports14040139

**Published:** 2026-04-01

**Authors:** María del Carmen Carcelén-Fraile, María Luisa Montánchez-Torres, Daniela Cecic-Mladinic

**Affiliations:** 1 Department of Educational Sciences, Faculty of Social Sciences, University of Atlántico Medio, 35017 Las Palmas de Gran Canaria, Spaindaniela.cecic@pdi.atlanticomedio.es (D.C.-M.); 2 International Scientific Association on Innovation in Education and Health (ACIINES), 23007 Jaén, Spain; 3 International Network of Educational Law, 35017 Las Palmas de Gran Canaria, Spain; 4 Department of Educational Sciences, Faculty of Education, International University of La Rioja, 26006 Logroño, Spain

**Keywords:** physical exercise, attention, adolescents, cognitive performance, neurocognitive development, children, executive functions

## Abstract

Background: Physical exercise has been increasingly recognized as a potential strategy to enhance cognitive development during childhood and adolescence. Among cognitive functions, attention plays a critical role in academic performance, behavioral regulation, and information processing. However, evidence regarding the specific effects of physical exercise on attentional performance in youth remains heterogeneous. Objective: This systematic review aimed to examine the effects of physical exercise interventions on attentional performance in children and adolescents. Methods: A systematic literature search was conducted in PubMed, Web of Science, Scopus, and CINAHL databases. Randomized controlled trials evaluating the effects of physical exercise interventions on attentional outcomes in participants aged 8 to 17 years were included. Study selection followed PRISMA guidelines. Nine studies met the inclusion criteria and were analyzed qualitatively. Results: The included studies consistently reported improvements in attentional performance following physical exercise interventions. Positive effects were observed across several attentional domains, including concentration, selective attention, sustained attention, processing speed, and response accuracy. Both acute and chronic exercise programs demonstrated cognitive benefits, although longer interventions appeared to produce more stable improvements. Coordinative and cognitively demanding exercise modalities tended to generate greater attentional gains compared with traditional physical activity programs. Conclusions: Physical exercise appears to be an effective non-pharmacological intervention for enhancing attentional performance in children and adolescents. Structured and cognitively engaging exercise programs may provide additional benefits for attentional development. Further research is needed to determine optimal exercise characteristics and to clarify the neurophysiological mechanisms underlying exercise-related attentional improvements.

## 1. Introduction

Childhood represents a critical stage for the development of fundamental cognitive processes, including attentional control [1]. During this period, attentional capacities such as selective attention, sustained attention, and attentional shifting progressively improve as a result of brain maturation and increasing environmental demands [2]. Neurodevelopmental research indicates that early attentional skills are closely linked to the development of executive functions and academic learning, particularly in areas such as reading, problem solving, and behavioral self-regulation [3]. These early attentional processes provide the cognitive foundation upon which more complex cognitive abilities continue to develop during adolescence. Adolescence represents a developmental period characterized by significant biological, psychological, and social transformations that directly influence the reorganization and maturation of the central nervous system [4,5]. During this stage, the development of higher cognitive functions becomes especially relevant, as these skills allow individuals to adapt to increasingly complex academic, social, and emotional demands [6]. Among these functions, attention is an essential cognitive process that enables the selection, focus, and maintenance of relevant information, as well as the inhibition of distracting stimuli [7]. The proper regulation of attentional processes is fundamental for learning, problem solving, behavioral control, and academic performance [8,9].

From a neuropsychological perspective, the development of attention during adolescence is closely associated with the progressive maturation of brain structures such as the prefrontal cortex, the parietal system, and the frontoparietal networks involved in cognitive control [10]. This maturational process promotes improved sustained attention, selective attention, and the ability to shift attentional focus between different tasks [11]. However, this stage is also characterized by a high vulnerability to environmental and behavioral factors that can influence the efficiency of attentional mechanisms [12].

In recent decades, various changes in the lifestyles of the adolescent population have generated concern regarding cognitive development [13]. The increase in sedentary behavior, the reduction in levels of physical activity, the increase in screen time, and changes in sleep patterns have been identified as factors that can interfere with the ability to concentrate and with attentional self-regulation [14]. These trends have driven the search for preventive and health promotion strategies that can support cognitive development during this stage of the life cycle [15].

In this context, physical exercise has been proposed as a potentially effective intervention to optimize brain and cognitive function [16]. Several theoretical models suggest that physical activity can influence cognitive processes through multiple neurobiological and psychological mechanisms [17]. Among these benefits are increased cerebral blood flow, the release of neurotransmitters related to attentional regulation, the stimulation of neurotrophic factors associated with neuronal plasticity, and improved functional connectivity between brain regions involved in cognitive control [18]. Furthermore, exercise can promote psychological processes such as emotional regulation, motivation, and physiological arousal, all of which have been shown to play a significant role in attentional performance [19,20].

Scientific interest in analyzing the relationship between physical exercise and cognitive function has increased considerably in recent years [21]. Numerous studies have examined the impact of different types of exercise on various cognitive functions, including working memory, inhibitory control, cognitive flexibility, and processing speed [22,23]. However, attention, despite being a fundamental component of the executive system and a prerequisite for the proper functioning of other cognitive processes, has frequently been evaluated as part of global cognitive constructs, which limits the specific understanding of its relationship with physical activity [24].

In addition, the existing literature exhibits considerable methodological heterogeneity [25]. Research differs in terms of the type of exercise applied, the intensity and duration of the interventions, the characteristics of the samples, and the instruments used to assess attentional processes [26,27]. These variations make it difficult to compare studies and generate results that, in some cases, show consistent positive effects, while in others they show limited or inconclusive effects [28]. This situation highlights the need for systematic reviews that allow for the integration and critical analysis of the available evidence [29].

On the other hand, it is especially relevant to focus the analysis on the adolescent population, given that this period constitutes a key window of opportunity for the development of cognitive functions and for the acquisition of healthy habits that can be maintained into adulthood [30]. Identifying effective interventions aimed at improving attentional performance could have important implications in the educational, sports, and healthcare fields, contributing to the design of programs that promote cognitive development and psychological well-being [31]. Given these premises, a rigorous synthesis of the scientific evidence is necessary to specifically examine the effect of exercise-based interventions on attentional performance in children and adolescents [32].

Consequently, the objective of this study is to systematically analyze the available scientific evidence on the effect of physical exercise on attentional performance in children and adolescents by reviewing experimental studies that evaluate this relationship.

## 2. Materials and Methods

### 2.1. Information Sources

A systematic search of the scientific literature was conducted to identify experimental studies analyzing the effect of physical exercise on attentional performance in adolescents. The search was performed in the electronic databases PubMed, Web of Science, Scopus, and CINAHL, selected for their broad coverage in the fields of psychology, neuroscience, sports science, and education. The search was conducted in February 2026. No initial restrictions were placed on the country where the studies were conducted. In addition, a manual review of the reference lists of the selected articles and of related systematic reviews was carried out to identify potentially relevant studies that had not been detected in the electronic search. This systematic review was registered in the PROSPERO database under the registration number CRD420261305299. The review was conducted in accordance with the registered protocol, and no deviations from the original PROSPERO protocol occurred during the review process.

### 2.2. Search Strategy

The search strategy was developed by combining terms related to physical exercise, attention, and adolescents. Boolean operators (AND, OR) were used, and the terms were adapted to the specific descriptors of each database. The general search string used was as follows: (“physical activity” OR “exercise” OR “physical training” OR “aerobic exercise” OR “motor intervention”) AND (“attention” OR “attentional performance” OR “sustained attention” OR “selective attention” OR “cognitive attention”) AND (“adolescent” OR “young adult” OR “adolescent”). Where possible, filters related to participant age, type of experimental study, and studies conducted in human populations were applied.

### 2.3. Inclusion Criteria

The following selected studies had to meet the criteria: (i) Experimental or quasi-experimental designs evaluating interventions based on physical exercise; (ii) participants aged between 8 and 17 years; (iii) interventions focused exclusively on structured physical exercise programs or planned physical activity; (iv) evaluation of outcomes specifically related to attentional processes, including sustained, selective, divided, or attentional control; (v) use of neuropsychological instruments, cognitive tests, or validated questionnaires for measuring attention.

### 2.4. Exclusion Criteria

Studies with any of the following characteristics were excluded: (i) observational studies without experimental intervention; (ii) studies that evaluate global cognitive functions without reporting specific attention-related outcomes; (iii) interventions that combine physical exercise with pharmacological, psychological, or other therapies without allowing isolation of the effect of exercise; (iv) studies conducted in adult or child populations outside the established age range; (v) review articles, case studies, editorials, or brief communications.

### 2.5. Study Selection Process

The selection process was carried out following the guidelines established by the PRISMA statement. In the first phase, duplicate records from the various databases were removed. Subsequently, titles and abstracts were reviewed to identify potentially relevant studies based on the eligibility criteria. Articles that met the initial criteria were assessed through full-text reading to confirm their inclusion. This procedure was carried out independently by two researchers. Any discrepancies were resolved by consensus with a third reviewer.

### 2.6. Data Extraction

Data extraction was carried out systematically to collect relevant information from each of the studies included in the review. A data collection form was designed to gather variables related to the general characteristics of the studies, including authorship, year of publication, and country of origin. Methodological aspects such as experimental design, sample size, mean age of participants, and the distribution of experimental and control groups were also recorded. Regarding the interventions, data were extracted on the type of physical exercise program applied, its total duration, weekly frequency, intensity, and application context. Information was also collected on the instruments used to assess attentional processes, as well as the specific domains of attention analyzed, such as sustained attention, selective attention, and attentional control. Finally, the main results obtained in relation to attentional performance were recorded, including the changes observed after the intervention and the statistical data reported by the authors. The data extraction process was performed independently by two researchers, and any discrepancies were resolved by consensus to ensure the reliability of the collected information.

### 2.7. Data Synthesis

A quantitative meta-analysis was initially considered during the review process. However, due to substantial heterogeneity across studies in terms of intervention characteristics (acute vs. chronic exercise, exercise modality, intensity and duration), comparator conditions, and the wide variety of neuropsychological instruments used to assess different attentional domains (e.g., d2 Test, Stroop Test, Trail Making Test, Letter Cancelation Test), pooling effect sizes was considered methodologically inappropriate. Therefore, a qualitative synthesis of the evidence was conducted.

### 2.8. Methodological Quality Assessment

The methodological quality of the studies included in this review was assessed using the PEDro scale, a widely used tool for evaluating clinical trials in research related to exercise-based interventions [33]. This scale allows for the analysis of fundamental aspects of experimental design, such as the randomization of participants, initial comparability between groups, blinding procedures, participant follow-up, statistical analysis, and the appropriate presentation of results. The total score on the scale ranges from 0 to 10 points, allowing studies to be classified into different levels of methodological quality: low quality (0–3 points), moderate quality (4–5 points), good quality (6–8 points), and excellent quality (9–10 points) [34]. The quality assessment was carried out independently by two reviewers with experience in scientific research, and any discrepancies were resolved by consensus to ensure the objectivity and rigor of the process.

## 3. Results

### 3.1. Study Selection Process

**Figure 1:**
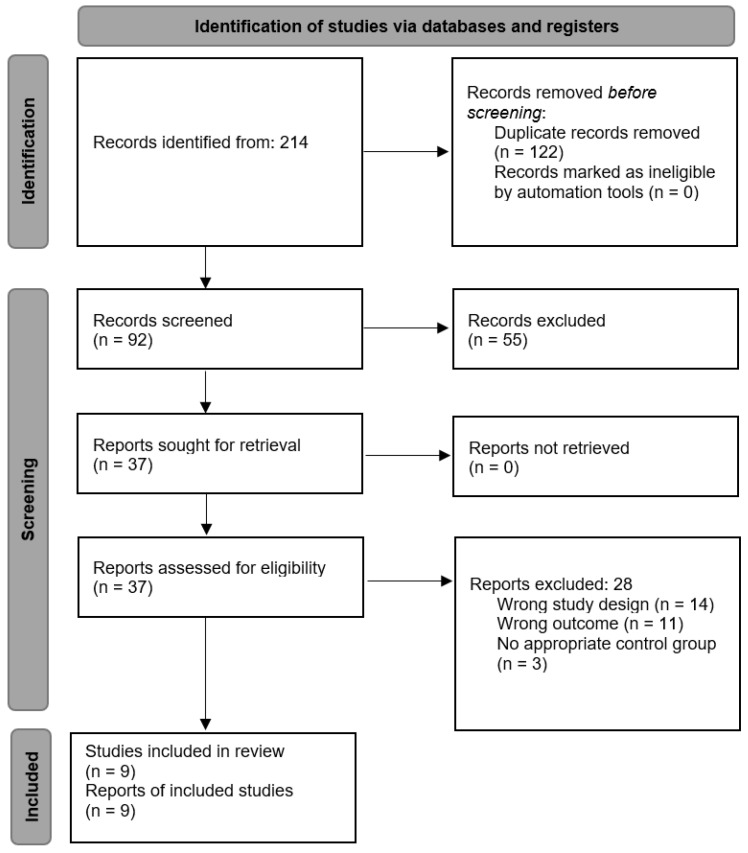
Study selection process flow chart.

A total of 214 records were initially identified through searches conducted across the selected electronic databases. Following the application of predefined filters related to study design (randomized controlled trials), publication language (English and Spanish), and target population (children and adolescents), 146 records remained. After removing duplicate entries, 92 unique studies were retained for further screening. During the initial screening phase, titles and abstracts were reviewed to determine their relevance to the research question. This process resulted in the exclusion of 55 studies that did not meet the inclusion criteria, primarily due to the absence of physical exercise interventions or lack of specific assessment of attentional outcomes. Consequently, 37 articles were considered eligible for full-text evaluation. Following full-text analysis, 28 studies were excluded for reasons including inappropriate study design, evaluation of global cognitive performance without reporting specific attentional measures, inclusion of adult populations, or combined interventions that did not allow isolation of the effects of physical exercise. Ultimately, 9 studies met all eligibility criteria and were included in the qualitative synthesis of this systematic review. The study selection process was conducted in accordance with the PRISMA 2020 guidelines (Appendix A) and is summarized in Figure 1.

### 3.2. Study Selection Process

The methodological quality of the included studies was assessed using the PEDro scale, the results of which are shown in Table 1. Scores ranged from 4 to 7 out of a maximum of 10, with an overall mean of 5.33, indicating moderate overall methodological quality. Most of the included studies scored 5/10, such as the works by Altermann and Gröpel [35], Subramanian et al. [36] and Ranjani et al. [37] achieved scores of 6/10, demonstrating adequate experimental design and randomization procedures, although with limitations in aspects related to blinding and complete follow-up of participants. Similarly, Gallotta et al. [38], Budde et al. [39], Vhavle et al. [40], and Altenburg et al. [41] also obtained scores of 5/10. In these cases, although randomized experimental designs were used and statistical comparisons between groups were performed with adequate reporting of results, they presented methodological limitations, mainly related to the lack of blinding of participants, therapists, and evaluators, as well as the lack of information on allocation concealment. The study with the highest methodological quality was that of Telles et al. [42], which scored 7/10, standing out for its adequate allocation concealment procedures, baseline similarity between groups, and blinding of evaluators. The study with the lowest score was that of Da Silva et al. [43], which obtained 4/10, mainly due to high loss to follow-up and the absence of intention-to-treat analysis, which could increase the risk of bias in the estimation of results. Overall, all studies met the criteria for randomization, statistical comparison between groups, and presentation of point and variability measures. However, none of the studies managed to blind participants or therapists, which is expected given the nature of exercise-based interventions. Likewise, blinding evaluators and concealing the allocation were criteria that were less frequently met.

### 3.3. Characteristics of the Included Studies

A total of nine randomized controlled trials were included in this systematic review. The studies were conducted across diverse geographical contexts, including Austria [32], India [36,37,42], Australia [40], Germany [39], Brazil [43], Italy [38] and the Netherlands [41]. All investigations examined the effects of structured physical activity or exercise-based interventions on attentional performance among children and adolescents. Across the included studies, participants ranged in age from 8 to 17 years. The total number of participants varied substantially between studies, from small experimental samples such as Da Silva et al. [43], which included 20 participants who completed the intervention, to large cluster trials such as Ranjani et al. [37], which involved approximately 2000 adolescents. Several studies reported balanced or mixed gender distributions, although some investigations did not specify detailed sex distribution [37,40]. The exercise interventions demonstrated considerable heterogeneity in design, modality, and duration. Some studies examined acute exercise effects, such as Budde et al. [39], Vhavle et al. [40] and Altenburg et al. [41], which implemented single-session interventions lasting between 10 and approximately 20 min. Notably, Altenburg et al. [41] investigated the differential effects of one versus two moderate-intensity physical activity bouts performed during a school morning, providing additional evidence regarding the influence of exercise frequency on attentional outcomes. In contrast, other investigations implemented longitudinal training programs ranging from 8 weeks to 6 months. For example, Da Silva et al. [43] applied an 8-week swimming training program with two weekly sessions, whereas Subramanian et al. [36] conducted a 6-month structured physical activity intervention consisting of six weekly sessions lasting approximately two hours each. Similarly, Gallotta et al. [38] implemented a 5-month school-based physical activity program with two weekly sessions of 60 min. The types of physical activity interventions also varied considerably across studies. Aerobic and multicomponent exercise programs were commonly implemented, including high-intensity interval training, strength circuits, and coordinative training as reported by Altermann and Gröpel [35]. Several studies emphasized structured physical activity programs designed according to international guidelines or educational curricula [36,38]. Other interventions focused on specific activity modalities such as swimming training [43] or yoga-based programs integrating breathing exercises, postures, meditation, and relaxation techniques [36,38]. Additionally, Budde et al. [39] specifically evaluated coordinative exercise protocols requiring complex motor control, balance, and reaction tasks.

Control conditions varied across studies but generally consisted of either usual school activities, non-structured physical education lessons, educational programs, or no additional intervention. For instance, Altermann and Gröpel [35] used regular school activities as a control condition, whereas Vhavle et al. [40] implemented a non-exercise indoor control group. Similarly, Altenburg et al. [41] used a seated classroom-based condition as the control group when examining the effects of one or two exercise bouts. In other studies, such as Subramanian et al. [36], comparisons were performed between structured and unstructured physical activity programs rather than between exercise and non-exercise conditions. Attentional performance was assessed using a variety of validated neuropsychological instruments. The d2 Test of Attention and its revised version were among the most frequently used measures [35,38,39]. Other studies employed tasks targeting different attentional domains, including the Stroop Color-Word Test and teacher-based attention ratings [36], the Letter Cancelation Test [36,37], and computerized cognitive assessments such as Rapid Visual Information Processing and Spatial Working Memory tasks [40]. Additionally, Da Silva et al. [43] used the Cancelation Attention Test combined with cognitive flexibility assessments, whereas Altenburg et al. [41] assessed selective attention using the Sky Search subtest from the Test of Everyday Attention for Children (Table 2).

Overall, the findings across studies consistently indicated beneficial effects of physical exercise interventions on attentional performance. Improvements were reported in several attentional domains, including concentration, processing speed, selective attention, sustained attention, and error reduction. Some studies suggested that specific exercise modalities may produce differential cognitive benefits. For example, coordinative training interventions were associated with greater improvements in concentration accuracy [38,39], while structured physical activity programs demonstrated superior gains in processing speed and task completion efficiency [36]. Similarly, yoga-based interventions showed improvements in attentional control and concentration compared with educational control conditions [37,42]. Moreover, Altenburg et al. [41] demonstrated that repeated moderate-intensity physical activity bouts during the school day may produce greater improvements in selective attention than a single exercise bout, highlighting the potential importance of exercise frequency in cognitive enhancement.

### 3.4. Study Results

All included studies reported outcomes related to attentional performance following physical exercise interventions. Although the interventions differed substantially in duration, intensity, and modality, most studies demonstrated improvements in at least one attentional domain. The studies included in this review covered a relatively broad developmental range, with participants aged between 8 and 17 years, encompassing both late childhood and adolescence.

Several studies evaluating chronic physical activity interventions reported consistent positive effects on attention. For example, Subramanian et al. [36] studied adolescents aged approximately 12–17 years and observed significant improvements in attention-related cognitive performance following both structured and unstructured physical activity programs, with greater benefits reported for structured interventions, particularly in processing speed and task completion. Similarly, Gallotta et al. [38] examined children aged approximately 8–11 years and found that both traditional and coordinative physical activity interventions enhanced attentional performance, although coordinative training produced greater improvements in concentration accuracy, whereas traditional physical activity improved processing speed. Comparable findings were reported by Ranjani et al. [37], who included adolescents aged 13–15 years, demonstrating significant increases in attention and concentration following a school-based yoga intervention, with improvements exceeding those observed in the educational control condition.

Additional evidence supporting long-term exercise effects was provided by Da Silva et al. [43], who investigated children aged approximately 11–14 years, reporting significant improvements in selective attention and cognitive flexibility following an eight-week swimming training program, while the control group showed no comparable changes. Likewise, Telles et al. [42], who examined children with a mean age of approximately 10.5 years, reported improvements in attentional control and interference processing following both yoga and physical exercise interventions, as demonstrated by enhanced performance on Stroop-based measures and teacher-reported attention assessments.

Studies investigating acute exercise effects also demonstrated positive outcomes. Budde et al. [39], who studied adolescents aged approximately 13–16 years, found that a single session of coordinative exercise significantly improved concentration, processing speed, and error reduction compared with standard physical education activities. Similarly, Altermann and Gröpel [35], whose sample consisted of adolescents with a mean age of approximately 16.5 years, reported that acute exercise interventions including high-intensity interval training, strength circuit training, and coordinative training improved attentional performance, with participants demonstrating enhanced concentration and information processing accuracy compared with baseline values. Furthermore, Altenburg et al. [41], who examined children aged 10–13 years, demonstrated that repeated moderate-intensity physical activity bouts performed during a school morning significantly improved selective attention compared with both a single exercise bout and a sedentary control condition, suggesting that exercise frequency may play an important role in optimizing attentional outcomes.

However, not all acute interventions produced consistent effects across conditions. Vhavle et al. [40], who included adolescents with a mean age of approximately 14.3 years, observed improvements in sustained attention accuracy following exercise sessions, but no significant differences were found between exercise environments, suggesting that contextual environmental factors may have limited influence on attentional outcomes.

Overall, the findings suggest that physical exercise interventions contribute to improvements in multiple attentional domains across both childhood and adolescence, including sustained attention, selective attention, concentration performance, processing speed, and response accuracy. In addition, several studies indicated that exercise modality may influence the magnitude and type of attentional benefits, with coordinative or cognitively demanding motor activities frequently demonstrating superior effects on concentration and attentional control compared with more traditional aerobic or general physical activity interventions. Moreover, evidence suggests that not only exercise type but also exercise frequency during the school day may influence attentional performance.

## 4. Discussion

The present systematic review aimed to examine the effects of physical exercise interventions on attentional performance in children and adolescents. Overall, the findings indicate that physical exercise represents an effective non-pharmacological strategy for improving attentional functioning across different developmental stages. Despite the variability observed in intervention characteristics, duration, and exercise modalities, most included studies reported improvements in attentional domains such as concentration, processing speed, sustained attention, and selective attention.

One of the most consistent findings across studies is the positive impact of both acute and chronic exercise interventions on attentional performance [44]. Acute exercise protocols demonstrated immediate cognitive benefits, particularly in tasks requiring rapid information processing and attentional accuracy [45]. For example, coordinative exercise interventions were shown to enhance concentration and reduce error rates shortly after exercise sessions [46]. These findings are consistent with theoretical models proposing that short-term exercise may induce transient increases in cortical activation, cerebral blood flow, and neurotransmitter release. Although these mechanisms were not directly measured in the studies included in this review, previous research has suggested that such neurophysiological responses may contribute to improvements in attentional regulation and executive control processes [7]. Emerging evidence also suggests that not only exercise modality and duration but also the distribution of exercise bouts throughout the school day may influence attentional outcomes. Recent findings indicate that repeated moderate-intensity exercise bouts may produce greater improvements in selective attention compared with a single exercise session, highlighting the importance of exercise frequency in sustaining acute cognitive benefits.

Chronic exercise interventions, on the other hand, appeared to produce more stable and long-lasting attentional improvements [47]. Longitudinal programs lasting several weeks or months consistently reported significant enhancements in cognitive performance [48]. These improvements may be explained by structural and functional neuroadaptations associated with repeated physical activity exposure [49]. Previous research suggests that regular exercise may promote neuroplasticity, increase brain-derived neurotrophic factor (BDNF) levels, and enhance synaptic connectivity within frontoparietal networks responsible for attentional control. However, it should be noted that the studies included in this review did not directly assess these neurobiological mechanisms, and therefore these explanations should be interpreted as theoretical frameworks derived from the broader literature. Such adaptations may facilitate more efficient neural processing and cognitive resource allocation, particularly during adolescence, a developmental period characterized by ongoing maturation of prefrontal cortical regions [18,50]. Furthermore, repeated exposure to exercise stimuli may contribute to maintaining neurophysiological activation across the school day, potentially extending the duration of cognitive benefits typically observed following single acute exercise sessions. Therefore, while these neurobiological mechanisms provide a plausible explanatory framework for the cognitive benefits of exercise, future studies should incorporate neurophysiological and neuroimaging measures to directly examine the mechanisms underlying exercise-induced improvements in attentional performance.

Another relevant finding of this review is the potential influence of exercise modality on attentional outcomes. Coordinative and cognitively demanding motor activities appeared to produce greater improvements in concentration accuracy and attentional control compared with traditional aerobic exercise programs [51]. These findings align with the hypothesis that complex motor tasks requiring decision-making, spatial orientation, and motor planning simultaneously stimulate multiple neural networks, including cerebellar and prefrontal regions. The integration of cognitive and motor demands during coordinative exercise may therefore enhance neural efficiency and promote executive functioning development [52].

Similarly, structured physical activity programs based on educational or clinical guidelines also demonstrated significant cognitive benefits, particularly in improving processing speed and task completion efficiency [53]. Structured interventions often provide progressive overload, task variation, and repeated cognitive–motor engagement, which may contribute to enhanced attentional performance [54]. Additionally, exercise programs such as swimming training and yoga-based interventions showed positive effects on attentional functioning [55]. Yoga interventions, in particular, may combine physical movement with breathing control, relaxation techniques, and mindfulness elements, which have been associated with improved attentional regulation and emotional control [56].

Another important aspect highlighted by this review is the effectiveness of physical exercise in diverse populations. Improvements in attentional performance were observed not only in typically developing children but also in participants with specific cognitive or behavioral characteristics, such as those with attention-deficit hyperactivity disorder [43]. Studies conducted in large and heterogeneous school samples have also demonstrated consistent benefits of physical exercise on attention [36,37,38,42]. This suggests that physical exercise interventions could represent a universally applicable strategy for promoting cognitive development, with potential implications for educational and clinical settings.

From an applied perspective, the results of this review support the integration of structured physical activity programs into school curricula and youth health promotion strategies. Exercise interventions that incorporate coordinative, cognitively demanding, and varied motor tasks may be particularly effective in enhancing attentional performance and academic readiness [57]. Schools represent an optimal environment for implementing such interventions due to their accessibility, structured routines, and potential for long-term behavioral habit formation [58]. In addition, the available evidence suggests that distributing multiple short physical activity breaks throughout the school day may enhance attentional regulation more effectively than isolated exercise sessions, supporting the implementation of active breaks within academic schedules [59]. From a practical perspective, these findings suggest that schools could incorporate short bouts of moderate physical activity (e.g., 10–20 min) before or between academic lessons to promote attentional readiness. Teachers may also integrate coordinative or cognitively engaging motor tasks within physical education classes or classroom-based active breaks to stimulate both motor and cognitive engagement. At the policy level, educational institutions could consider promoting school-wide physical activity programs or structured movement breaks as part of strategies aimed at improving students’ cognitive functioning, classroom engagement, and learning outcomes.

Despite these promising findings, several limitations should be considered when interpreting the results. First, considerable heterogeneity was observed across studies in terms of intervention characteristics, exercise intensity, duration, and assessment tools used to measure attention. This variability may limit direct comparisons between studies and complicate the identification of optimal exercise protocols. Second, some studies included relatively small sample sizes, which may reduce statistical power and generalizability. Third, the use of different neuropsychological instruments across studies introduces variability in attentional domain assessment, potentially affecting the consistency of reported outcomes. Additionally, variability in exercise frequency and distribution across studies limits the ability to determine optimal scheduling strategies for maximizing attentional benefits. These sources of heterogeneity also limited the feasibility of conducting a robust quantitative meta-analysis across the included trials.

In addition to the variability in intervention protocols and outcome measures, the methodological quality assessment revealed several potential sources of bias across the included trials. Although most studies used randomized designs and reported appropriate statistical comparisons, several methodological limitations were identified, particularly related to the absence of participant and therapist blinding, limited reporting of allocation concealment, and incomplete follow-up in some studies. While blinding procedures are often difficult to implement in exercise-based interventions, these factors may increase the risk of performance and detection bias and should be considered when interpreting the reported cognitive effects.

Furthermore, although randomized controlled trials provide strong methodological evidence, future research should incorporate longitudinal follow-up designs to determine the persistence of exercise-induced attentional benefits over time. Additional studies examining dose–response relationships, exercise intensity thresholds, and neurophysiological mechanisms underlying cognitive improvements are also warranted. The integration of neuroimaging and neurobiological markers may provide deeper insights into the mechanisms linking physical exercise and attentional development.

## 5. Conclusions

The findings of this systematic review indicate that physical exercise is an effective non-pharmacological strategy for improving attentional performance in children and adolescents. Across the included studies, exercise interventions demonstrated consistent positive effects on several attentional domains, including concentration, selective attention, sustained attention, processing speed, and response accuracy. Both acute and chronic exercise programs were associated with cognitive improvements, although longer interventions appeared to produce more stable and sustained attentional benefits. In addition, emerging evidence suggests that the frequency and distribution of exercise bouts throughout the school day may influence the magnitude and duration of attentional improvements, with repeated short exercise sessions potentially providing greater cognitive benefits than isolated activity bouts. In particular, studies examining multiple exercise bouts within a school morning reported greater improvements in selective attention compared with single exercise sessions, highlighting the potential importance of exercise scheduling in educational contexts. Furthermore, the results suggest that exercise modality may play a relevant role in attentional development. Interventions incorporating coordinative, structured, and cognitively engaging motor activities tended to produce greater improvements in attentional control compared with traditional physical activity programs. Taken together, the available evidence indicates that exercise modality, duration, and frequency interact to determine attentional outcomes in youth populations. These findings highlight the potential value of integrating structured exercise programs into educational and youth health settings as a strategy to support cognitive development and academic performance. Educational strategies incorporating multiple short physical activity breaks during the school day may represent an effective approach to optimize attentional regulation and learning readiness. Future research should focus on identifying optimal exercise parameters and clarifying the neurophysiological mechanisms underlying exercise-induced attentional improvements. Further investigations examining dose–response relationships, exercise scheduling, and long-term cognitive retention are also warranted.

## Figures and Tables

**Table 1:** Methodological quality of the included articles.

Study	1	2	3	4	5	6	7	8	9	10	11	Total (0–10)
Altermann and Gröpel [35]	1	1	0	1	0	0	0	1	0	1	1	5
Subramanian et al. [36]	1	1	1	1	0	0	1	0	0	1	1	6
Ranjani et al. [37]	1	1	1	0	0	0	0	1	1	1	1	6
Gallotta et al. [38]	1	1	0	1	0	0	0	1	0	1	1	5
Telles et al. [42]	1	1	1	1	0	0	1	1	0	1	1	7
Budde et al. [39]	1	1	0	1	0	0	0	1	0	1	1	5
Da Silva et al. [43]	1	1	0	1	0	0	0	0	0	1	1	4
Vhavle et al. [40]	1	1	0	0	0	0	0	1	1	1	1	5
Altenburg et al. [41]	1	1	0	1	0	0	0	1	0	1	1	5

Scoring criteria: (1) eligibility specified; (2) random allocation; (3) concealed allocation; (4) comparable groups at baseline; (5) participant blinding; (6) therapist blinding; (7) assessor blinding; (8) adequate follow-up; (9) intention-to-treat analysis; (10) between-group statistical comparisons. Each item is scored as Yes = 1 or No = 0.

**Table 2:** **Summary of the characteristics of the included studies.**

Authors	Country	Study Design	Age	Participants (M/F)	Sample Size (N)	Control Group	Intervention Group	Session Duration	Session Frequency	Outcome Measures	Main Results
Altermann and Gröpel [35]	Austria	RCT	16.5 ± 1.12	80 (39/41)	IG = 60; CG = 20	Regular school activities/no structured physical training intervention	High-intensity interval training (HIIT), bodyweight strength circuit, and coordinative training	25 min	Not specified	D2 Test of Attention	Physical exercise interventions were associated with improvements in attentional performance, reflected by enhanced concentration and processing accuracy compared to baseline values, while control participants showed no comparable improvement.
Subramanian et al. [36]	India	RCT	12–17 years (median ≈ 14 years)	439 recruited (250 males/189 females); 347 completed	SPA = 136; USPA = 139 (per-protocol)	Unstructured physical activity (USPA): supervised recreational and sport activities freely chosen by participants	Structured physical activity (SPA) based on WHO guidelines including aerobic, muscle-strengthening, stretching, and sport-based activities	Approximately 2 h per session	6 sessions/week for 6 months	Digit Span Test (Wechsler Intelligence Scale) Trail Making Test A and B (TMT-A/B)	Both structured and unstructured physical activity significantly improved attention-related cognitive performance. However, structured physical activity produced greater improvements in attentional measures, particularly processing speed and task completion time.
Ranjani et al. [37]	India	RCT	13–15 years	Not specified	2000 participants	Education-based healthy lifestyle program including monthly awareness sessions and stretching exercises	Standardized school-based yoga program including pranayama, yoga postures, meditation, relaxation exercises, and reflective discussions	~45 min	17 sessions over approximately 5–6 months (≈once per week)	Stroop Color-Word Test	The yoga intervention significantly improved attention and concentration performance, showing an 18% increase in LCT scores compared with a 7% increase in the control group.
Gallotta et al. [38]	Italy	RCT	8–11 years	Not fully specified (Normal weight and overweight/obese children included)	156 participants	Control group not attending any structured physical activity program	Two physical activity interventions: Traditional physical activity (endurance, strength, flexibility, circuit training) and Coordinative physical activity (sports games, rhythmic activities, gymnastics, and fitness-based coordinative exercises)	60 min	2 sessions/week for 5 months	D2 Test of Attention	Both physical activity interventions significantly improved attention performance compared with control. Coordinative training produced greater improvements in concentration.
Telles et al. [42]	India	RCT	10.5 ± 1.3	98 total (60 boys/38 girls)	Yoga = 49; Physical Exercise = 49	Yoga intervention (comparison group for physical exercise analysis)	Physical exercise including jogging-in-place, rapid bending movements, spinal twisting, relay races, and games	45 min	5 sessions/week	Stroop Color–Word Test	Both yoga and physical exercise significantly improved Stroop task performance and teacher-rated attention scores after the intervention. Physical exercise also showed improvements in interference control measures, suggesting enhanced attentional processing and response inhibition.
Budde et al. [39]	Germany	RCT	13–16 years (Mean ≈ 15 years)	99 participants analyzed (80 males/19 females)	Experimental = 47; Control = 52	Normal sport lesson performed at moderate intensity without coordinative emphasis	Acute bilateral coordinative exercise involving balance, reaction, and multi-limb coordination tasks	10 min	Single acute session	d2 Test of Attention	Both exercise conditions improved attentional performance; however, coordinative exercise produced significantly greater improvements in concentration, processing speed, and error reduction compared with the control sport lesson.
Da Silva et al. [43]	Brazil	RCT	11–14 years	20 completed (Trained = 10; Control = 10)	Initially 33 participants	Untrained group maintaining usual activities without swimming training	Swimming-learning training program including aquatic adaptation exercises, propulsion drills, breathing training, and swimming coordination tasks	~45 min	2 sessions/week for 8 weeks (16 sessions total)	Cancelation Attention Test (TAC) Trail Making Test A and B (TMT-A/B)	Swimming training significantly improved selective attention and cognitive flexibility compared with baseline, while no significant changes were observed in the control group.
Vhavle et al. [40]	Australia	RCT	14.3 ± 0.05	Not specified	90 students	Non-exercise indoor control group	Exercise circuit including aerobic and bodyweight resistance activities performed in different environmental conditions (indoor, outdoor moderate nature, outdoor high nature)	~20 min	Single acute session	Trail Making Test A and B (TMT-A/B	Exercise sessions produced improvements in sustained attention accuracy in some exercise and control conditions; however, no significant differences were found between exercise environments, suggesting limited influence of environmental context on attentional outcomes.
Altenburg et al. [41]	Netherlands	RCT	10–13 years	56 (30 boys/26 girls)	56 participants	Seated classroom-based condition involving simulated school tasks without physical activity	Moderate-intensity aerobic physical activity using video-based dance exercises comparing one versus two exercise bouts	20 min	One or two acute sessions performed within a single school morning	d2 Test of Attention	Repeated moderate-intensity physical activity bouts significantly improved selective attention compared with a single exercise bout or sedentary control condition, suggesting that exercise frequency may influence attentional performance.

RCT: randomized controlled trial; IG: intervention group; CG: control group; SPA: structured physical activity; USPA: unstructured physical activity; HIIT: high-intensity interval training; TMT-A/B: Trail Making Test A and B; TAC: Cancelation Attention Test; Session duration refers to the length of each exercise session, while session frequency indicates the number of sessions performed within the intervention period.

## Data Availability

No new data were created or analyzed in this study.

## Abbreviations

The following abbreviations are used in this manuscript:

RCT	Randomized Controlled Trial
IG	Intervention Group
CG	Control Group
SPA	Structured Physical Activity
USPA	Unstructured Physical Activity
HIIT	High-Intensity Interval Training
TMT-A/B	Trail Making Test A and B
TAC	Cancelation Attention Test

## Appendix A. PRISMA Checklist

**Table:**

Section and Topic	Item #	Checklist Item	Location Where Item Is Reported
**TITLE**			
Title	1	Identify the report as a systematic review.	1
**ABSTRACT**			
Abstract	2	See the PRISMA 2020 for Abstracts checklist.	1
**INTRODUCTION**			
Rationale	3	Describe the rationale for the review in the context of existing knowledge.	2-3
Objectives	4	Provide an explicit statement of the objective(s) or question(s) the review addresses.	3
**METHODS**			
Eligibility criteria	5	Specify the inclusion and exclusion criteria for the review and how studies were grouped for the syntheses.	3-4
Information sources	6	Specify all databases, registers, websites, organisations, reference lists and other sources searched or consulted to identify studies. Specify the date when each source was last searched or consulted.	3
Search strategy	7	Present the full search strategies for all databases, registers and websites, including any filters and limits used.	3
Selection process	8	Specify the methods used to decide whether a study met the inclusion criteria of the review, including how many reviewers screened each record and each report retrieved, whether they worked independently, and if applicable, details of automation tools used in the process.	4
Data collection process	9	Specify the methods used to collect data from reports, including how many reviewers collected data from each report, whether they worked independently, any processes for obtaining or confirming data from study investigators, and if applicable, details of automation tools used in the process.	4
Data items	10a	List and define all outcomes for which data were sought. Specify whether all results that were compatible with each outcome domain in each study were sought (e.g., for all measures, time points, analyses), and if not, the methods used to decide which results to collect.	4
	10b	List and define all other variables for which data were sought (e.g., participant and intervention characteristics, funding sources). Describe any assumptions made about any missing or unclear information.	4
Study risk of bias assessment	11	Specify the methods used to assess risk of bias in the included studies, including details of the tool(s) used, how many reviewers assessed each study and whether they worked independently, and if applicable, details of automation tools used in the process.	4
Effect measures	12	Specify for each outcome the effect measure(s) (e.g., risk ratio, mean difference) used in the synthesis or presentation of results.	4
Synthesis methods	13a	Describe the processes used to decide which studies were eligible for each synthesis (e.g., tabulating the study intervention characteristics and comparing against the planned groups for each synthesis (item #5)).	3-4
	13b	Describe any methods required to prepare the data for presentation or synthesis, such as handling of missing summary statistics, or data conversions.	3-4
	13c	Describe any methods used to tabulate or visually display results of individual studies and syntheses.	3-4
	13d	Describe any methods used to synthesize results and provide a rationale for the choice(s). If meta-analysis was performed, describe the model(s), method(s) to identify the presence and extent of statistical heterogeneity, and software package(s) used.	3-4
	13e	Describe any methods used to explore possible causes of heterogeneity among study results (e.g., subgroup analysis, meta-regression).	3-4
	13f	Describe any sensitivity analyses conducted to assess robustness of the synthesized results.	3-4
Reporting bias assessment	14	Describe any methods used to assess risk of bias due to missing results in a synthesis (arising from reporting biases).	3-4
Certainty assessment	15	Describe any methods used to assess certainty (or confidence) in the body of evidence for an outcome.	3-4
**RESULTS**			
Study selection	16a	Describe the results of the search and selection process, from the number of records identified in the search to the number of studies included in the review, ideally using a flow diagram.	4-15
	16b	Cite studies that might appear to meet the inclusion criteria, but which were excluded, and explain why they were excluded.	4-5
Study characteristics	17	Cite each included study and present its characteristics.	5-12
Risk of bias in studies	18	Present assessments of risk of bias for each included study.	5-6
Results of individual studies	19	For all outcomes, present, for each study: (a) summary statistics for each group (where appropriate) and (b) an effect estimate and its precision (e.g., confidence/credible interval), ideally using structured tables or plots.	13
Results of syntheses	20a	For each synthesis, briefly summarise the characteristics and risk of bias among contributing studies.	13
	20b	Present results of all statistical syntheses conducted. If meta-analysis was done, present for each the summary estimate and its precision (e.g., confidence/credible interval) and measures of statistical heterogeneity. If comparing groups, describe the direction of the effect.	-
	20c	Present results of all investigations of possible causes of heterogeneity among study results.	5-12
	20d	Present results of all sensitivity analyses conducted to assess the robustness of the synthesized results.	5-12
Reporting biases	21	Present assessments of risk of bias due to missing results (arising from reporting biases) for each synthesis assessed.	5-12
Certainty of evidence	22	Present assessments of certainty (or confidence) in the body of evidence for each outcome assessed.	5-12
**DISCUSSION**			
Discussion	23a	Provide a general interpretation of the results in the context of other evidence.	14-15
	23b	Discuss any limitations of the evidence included in the review.	15
	23c	Discuss any limitations of the review processes used.	15
	23d	Discuss implications of the results for practice, policy, and future research.	15
**OTHER INFORMATION**			
Registration and protocol	24a	Provide registration information for the review, including register name and registration number, or state that the review was not registered.	3
	24b	Indicate where the review protocol can be accessed, or state that a protocol was not prepared.	3
	24c	Describe and explain any amendments to information provided at registration or in the protocol.	3
Support	25	Describe sources of financial or non-financial support for the review, and the role of the funders or sponsors in the review.	16
Competing interests	26	Declare any competing interests of review authors.	16
Availability of data, code and other materials	27	Report which of the following are publicly available and where they can be found: template data collection forms; data extracted from included studies; data used for all analyses; analytic code; any other materials used in the review.	16

*From*: ref. [60]. This work is licensed under CC BY 4.0. To view a copy of this license, visit https://creativecommons.org/licenses/by/4.0/.
